# Plasma lipocalin-2/NGAL is stable over 12 weeks and is not modulated by exercise or dieting

**DOI:** 10.1038/s41598-021-83472-x

**Published:** 2021-02-18

**Authors:** Michael E. Nakai, Joshua Denham, Priscilla R. Prestes, Nina Eikelis, Elisabeth A. Lambert, Nora E. Straznicky, Markus P. Schlaich, Murray D. Esler, Brendan J. O’Brien, Fadi J. Charchar, Gavin W. Lambert, Francine Z. Marques

**Affiliations:** 1 Hypertension Research Laboratory, School of Biological Sciences, Faculty of Science, Monash University, 25 Rainforest Walk, Melbourne, Clayton, VIC 3800 Australia; 2 Discipline of Exercise and Sports Science, School of Health and Biomedical Sciences, RMIT University, Melbourne, VIC 3083 Australia; 3 Faculty of Science and Technology, Federation University Australia, Mount Helen, Australia; 4 Iverson Health Innovation Research Institute and Faculty of Health, Arts and Design, Swinburne University of Technology, Melbourne, Australia; 5 Baker Heart and Diabetes Institute, Melbourne, Australia; 6 School of Medicine – Royal Perth Hospital Unit,Dobney Hypertension Centre, University of Western Australia, Perth, Australia

**Keywords:** Molecular biology, Cardiology, Nephrology

## Abstract

Amongst other immune cells, neutrophils play a key role in systemic inflammation leading to cardiovascular disease and can release inflammatory factors, including lipocalin-2 (LCN2). LCN2 drives cardiac hypertrophy and plays a role in maladaptive remodelling of the heart and has been associated with renal injury. While lifestyle factors such as diet and exercise are known to attenuate low-grade inflammation, their ability to modulate plasma LCN2 levels is unknown. Forty-eight endurance athletes and 52 controls (18–55 years) underwent measurement for various cardiovascular health indicators, along with plasma LCN2 concentration. No significant difference in LCN2 concentration was seen between the two groups. LCN2 was a very weak predictor or absent from models describing blood pressures or predicting athlete status. In another cohort, 57 non-diabetic overweight or obese men and post-menopausal women who fulfilled Adult Treatment Panel III metabolic syndrome criteria were randomly allocated into either a control, modified Dietary Approaches to Stop Hypertension (DASH) diet, or DASH and exercise group. Pre- and post-intervention demographic, cardiovascular health indicators, and plasma LCN2 expression were measured in each individual. While BMI fell in intervention groups, LCN2 levels remained unchanged within and between all groups, as illustrated by strong correlations between LCN2 concentrations pre- and 12 weeks post-intervention (*r* = 0.743, *P* < 0.0001). This suggests that circulating LCN2 expression are stable over a period of at least 12 weeks and is not modifiable by diet and exercise.

## Introduction

High body mass index (BMI) and obesity promote a low-grade chronic systemic inflammatory state, triggering a systemic acute phase response^1^. Pro-inflammatory acute phase response proteins are then produced and released, which can feedback into the chronic inflammatory phenotype^2^. Low-grade systemic inflammation also produces an ideal environment for the development of cardiovascular (CVD) and renal disease. Inflammatory biomarkers such as C-reactive protein (CRP) and the more recently implicated GlycA can predict the risk of CVD many years in advance^3,4^. Inflammatory pathways are critical for the pathological remodelling of the heart, for example, through stimulation of proteins involved in degradation and reconstruction of the interstitium^5–7^. This maladaptive remodelling is involved in multiple types of CVDs, and may lead to cardiac hypertrophy and heart failure^8^.

Neutrophils, the most abundant type of white blood cells in the body, are recognized for their role in the atherosclerotic process and their relevance for the development of heart failure^9,10^. Among a variety of inflammatory factors, neutrophils release lipocalin-2 (LCN2, otherwise known as neutrophil gelatinase-associated lipocalin or NGAL), a 25 kDa protein that is upregulated during the acute phase response, and primarily contributes to innate immunity through its function as an iron chelator^11,12^. It has been used clinically as a marker of renal failure and, more recently, as a marker of increased cardiovascular risk^13–15^. Other both immune and non-immune cell types such as cardiomyocytes, fibroendothelial cells, renal cells, and macrophages, have also been shown to release LCN2^16–19^. In the context of cardiac remodelling, LCN2 has been shown to preserve the enzymatic activity of matrix metalloprotease-9 (MMP9), a protein heavily involved in the breakdown of the interstitium^20,21^. LCN2 has also been shown to drive cardiac hypertrophy in vitro and *vivo*, and was associated with left ventricular mass and BMI in humans^19^. Importantly, LCN2 was a stronger marker of innate immune response, in particular neutrophil function, than CRP or GlycA^19^.

The ability of a balanced diet and regular exercise to reduce BMI and the risk of CVD is well documented^22–24^. In particular, specific diets such as the Dietary Approach to Stop Hypertension (DASH) have been able to reduce low-grade inflammation observed as serum CRP levels^25–29^. However, whether LCN2 expression can be attenuated by exercise or diet interventions is currently unclear. Here we aimed to determine if LCN2 plasma levels are modulated by physical fitness and diet, and to investigate whether LCN2 levels are stable over time.

## Results

**Figure 1:**
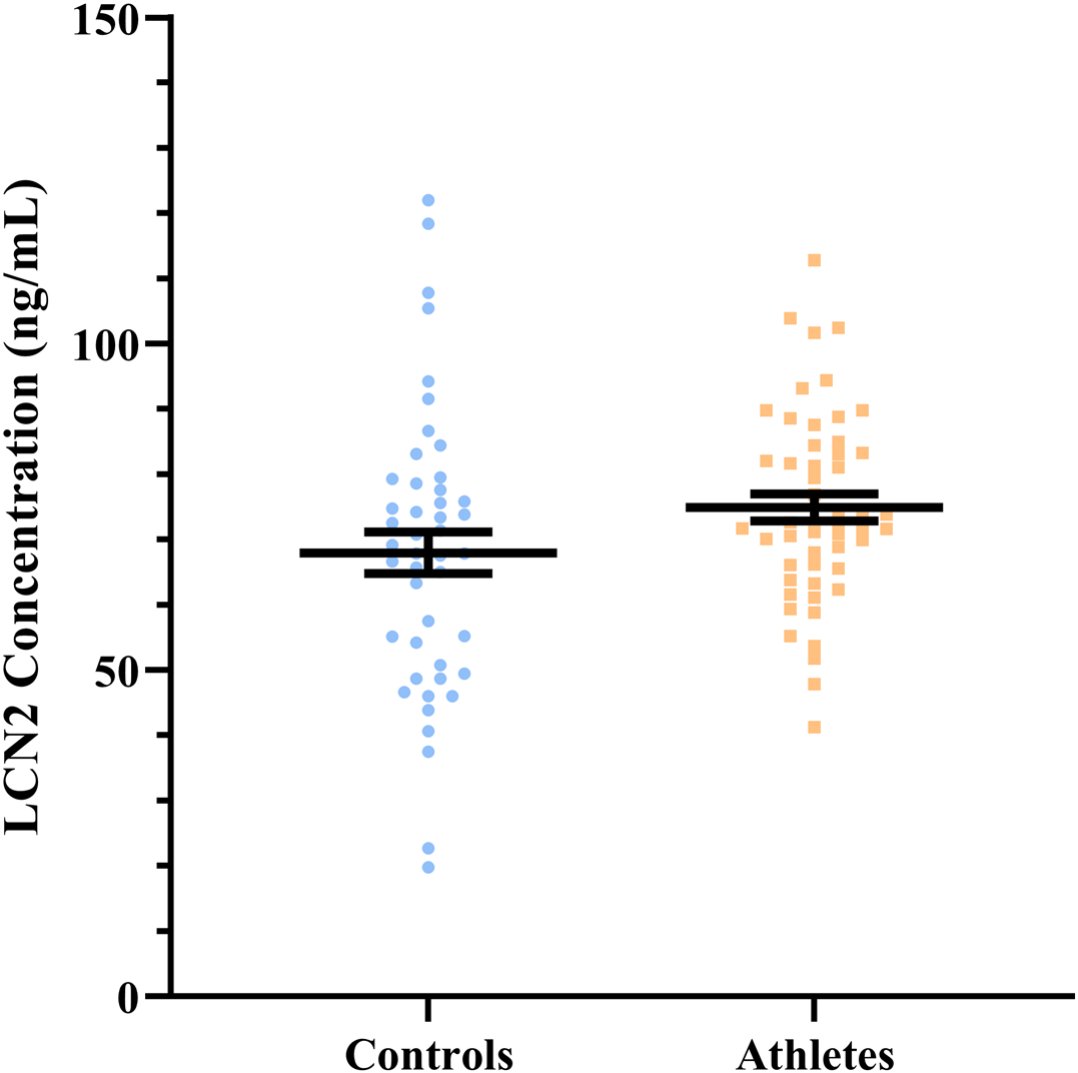
There was no difference in plasma lipocalin-2 (LCN2) protein levels between controls (n = 48) and endurance athletes (n = 52) (*P* = 0.073). Data are expressed as mean ± S.E.M. from two-tailed Man-Whitney U-test.

Relative to controls, endurance athletes were taller, had superior cardiorespiratory fitness as indicated by peak oxygen uptake ($\dot{V}$O_2peak_) (controls vs athletes mean ± SD, units: 42.6 ± 7.1 v 58.0 ± 8.8, ml^.^kg^−1^ min^−1^), lower resting heart rate (68 ± 10.5 v 52 ± 7.8, beats min^−1^), and BMI (25.9 ± 2.8 v 22.6 ± 2.3) (all *P* < 0.001, Table 1). The difference in plasma LCN2 protein levels between controls and endurance athletes was of borderline statistical significance (68.3 ± 21.8 v 75.0 ± 14.8, ng/mL, *P* = 0.073, Fig. 1). Therefore, a sensitivity analysis was performed to determine which cardiovascular health indicators could predict whether an individual belonged to the athlete group or control group (athlete status) (Table 2). The analysis showed that athlete status could be predicted correctly in 94.8% of individuals using BMI, sex, age, and $\dot{V}$O_2peak_ as predictors, with the model having an overall Cox & Snell *β* value of 0.683. LCN2 concentration, however, was not a significant predictor of athlete status in this model.

**Table 1:** Characteristics of endurance athletes and controls.

Variable	Controls (n = 48)	Endurance athletes (n = 52)	*P* value
Men/women (n)	34/14	41/11	0.36
Age (years)	30.5 ± 11.3	33.4 ± 11.1	0.19
BMI (kg/m^2^)	25.9 ± 2.8	22.6 ± 2.3	**< 0.001**
SBP (mmHg)	125.6 ± 10.0	122.6 ± 9.6	0.130
DBP (mmHg)	76.1 ± 8.8	72.2 ± 7.2	**0.018**
MAP (mmHg)	92.4 ± 8.3	88.9 ± 7.3	**0.030**
Central SBP (mmHg)	109.7 ± 9.1	105.0 ± 9.7	**0.016**
Central DBP (mmHg)	77.1 ± 8.7	72.6 ± 7.3	**0.007**
Central MAP (mmHg)	87.7 ± 8.8	84.8 ± 7.8	0.089
Resting HR (beats min^−1^)	68 ± 10.5	52 ± 7.8	**< 0.001**
$\dot{V}$O_2peak_ (ml kg^−1^ min^−1^)	42.6 ± 7.1	58.0 ± 8.8	**< 0.001**

Data are expressed as mean ± standard deviation from two-tailed independent samples *t*-tests or Mann–Whitney U-tests, with *P*-values below 0.05 bolded. Variances were equal between groups for all variables except for resting HR.

BMI, body mass index; SBP, systolic blood pressure; DBP, diastolic blood pressure; HR, resting heart rate; $\dot{V}$O_2peak_, maximal aerobic (cardiorespiratory) fitness.

**Table 2:** Results of sensitivity analysis performed on athlete status of an individual testing cohort demographic and cardiovascular health characteristics as well as LCN2 concentration.

Dependent variable	Cox & snell R^2^	Variables	*β*	*β* Std. Error	*P* value	Overall model
Athlete status	0.683	**Sex**	**− 5.511**	**2.8**	**0.049**	log(*p*/1 **− p**) = 0.361*Age + 0.74*$\dot{V}$O_2peak_ – 5.511 (Male) – 0.695*BMI
		**BMI**	− **0.695**	**0.35**	**0.047**	
		**Age**	**0.361**	**0.126**	**0.004**	
		$\dot{V}$**O**_**2peak**_	**0.74**	**0.242**	**0.002**	
		LCN2 Concentration	N/A	N/A	0.692	

Athletes were assigned as 1 in the dataset, while controls were assigned as 0. The overall model describes the relationship between significant predictors of athlete status and the predicted log odds of the individual tested being an athlete. The model correctly predicted athlete status in 94.8% of individuals in our dataset.

BMI, body mass index; SBP, systolic blood pressure; DBP, diastolic blood pressure; LCN2, lipocalin-2; $\dot{V}$O_2peak_ maximal aerobic (cardiorespiratory) fitness.

**Figure 2:**
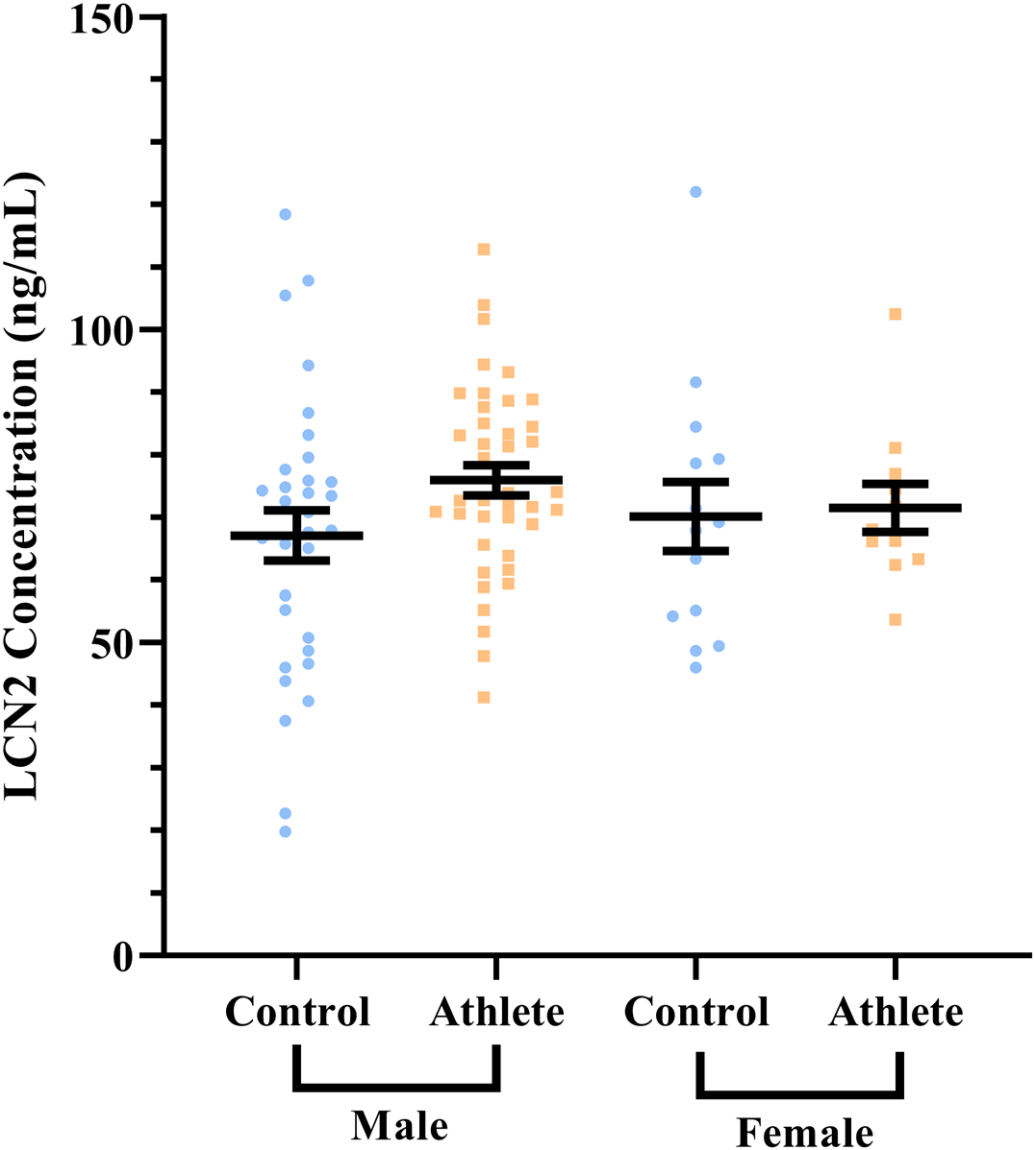
There was no significant difference in plasma lipocalin-2 (LCN2) protein levels between male controls (n = 34) and endurance athletes (n = 41) (*P* = 0.053), or between female controls (n = 14) and endurance athletes (n = 11) (*P* = 0.847). Data are expressed as mean ± S.E.M. from two-tailed Man-Whitney U-test.

To understand whether the borderline difference in plasma LCN2 expression could be explained through sex-specific differences, we analysed the cohort by splitting the controls and endurance athletes into male and female groups. Male athletes were more similar to sex-matched control group counterparts than when compared to female athletes compared to sex-matched controls in terms of cardiovascular health indicators (Tables 3, 4). Female athletes had a lower BMI (26.6 ± 3.4 v 20.9 ± 2.3), diastolic blood pressure (DBP) (76.6 ± 8.8 v 67.7 ± 7.4, mmHg), mean arterial pressure (MAP) (91.0 ± 9.3 v 82.7 ± 6.3, mmHg), central systolic blood pressure (SBP) (107.6 ± 11.3 v 96.9 ± 7.0, mmHg), central DBP (77.6 ± 8.8 v 67.8 ± 7.3, mmHg), central MAP (87.7 ± 9.3 v 77.5 ± 6.9, mmHg), resting heart rate (72.0 ± 9.6 v 52.5 ± 9.9, beats min^−1^), and higher $\dot{V}$O_2peak_ (34.7 ± 4.4 v 54.6 ± 13.1, ml kg^−1.^min^−1^), while male athletes only differed from the controls when looking at BMI (25.6 ± 2.5 v 23.0 ± 2.2), resting heart rate (67.2 ± 10.7 vs 52.3 ± 7.3, beats min^−1^), and $\dot{V}$O_2peak_ (45.8 ± 5.2 vs 59.0 ± 7.2, ml kg^−1^ min^−1^) (all *P* < 0.01). Importantly, the difference in plasma LCN2 expression was of borderline statistical significance in male controls compared to male athletes (67.1 ± 22.6 v 75.9 ± 15.3, ng/mL, *P* = 0.053), while there were no differences between controls and athletes in females (70.1 ± 20.6 v 71.5 ± 12.7, ng/mL, *P* = 0.847) (Fig. 2). Thus, a sensitivity analysis was again performed on the male data to test whether athlete status could be predicted from LCN2 expression, BMI, age, and $\dot{V}$O_2peak_ (Table 5). Male athlete status was found to be able to be predicted in 90.5% of the data using age and $\dot{V}$O_2peak_ as inputs, with the model showing an overall Cox & Snell *β* value of 0.676. LCN2 expression and BMI were not found to be significant predictors of athlete status. Therefore, males and females were grouped for all subsequent analyses.

**Table 3:** Characteristics of endurance athletes and controls in males.

Variable	Controls	Endurance athletes	*P* value
	(n = 34)	(n = 41)	
Age (years)	29.7 ± 10.4	33.6 ± 12.0	0.134
BMI (kg/m^2^)	25.6 ± 2.5	23.0 ± 2.2	**< 0.001**
SBP (mmHg)	128.0 ± 8.6	125.4 ± 8.3	0.189
DBP (mmHg)	75.9 ± 8.9	73.5 ± 6.7	0.193
MAP (mmHg)	93.0 ± 7.9	90.7 ± 6.6	0.189
Central SBP (mmHg)	110.6 ± 8.1	107.1 ± 9.2	0.107
Central DBP (mmHg)	76.9 ± 8.7	73.9 ± 6.8	0.105
Central MAP (mmHg)	87.6 ± 8.8	86.7 ± 7.0	0.611
Resting HR (beats min^−1^)	67.2 ± 10.7	52.3 ± 7.3	**< 0.001**
$\dot{V}$O_2peak_ (ml kg^−1^ min^−1^)	45.8 ± 5.2	59.0 ± 7.2	**< 0.001**

Data are expressed as mean ± standard deviation from two-tailed independent samples *t*-tests or Mann–Whitney U-tests, with *P*-values below 0.05 bolded. Variances were equal between groups for all variables except for resting HR and $\dot{V}$O_2peak_.

BMI, body mass index; SBP, systolic blood pressure; DBP, diastolic blood pressure; HR, resting heart rate; $\dot{V}$O_2peak_, maximal aerobic (cardiorespiratory) fitness.

**Table 4:** Characteristics of endurance athletes and controls in females.

Variable	Controls	Endurance athletes	*P* value
	(n = 14)	(n = 11)	
Age (years)	32.4 ± 13.5	32.7 ± 7.4	0.945
BMI (kg/m^2^)	26.6 ± 3.4	20.9 ± 2.3	**< 0.001**
SBP (mmHg)	119.9 ± 11.1	112.6 ± 7.1	0.071
DBP (mmHg)	76.6 ± 8.8	67.7 ± 7.4	**0.013**
MAP (mmHg)	91.0 ± 9.3	82.7 ± 6.3	**0.018**
Central SBP (mmHg)	107.6 ± 11.3	96.9 ± 7.0	**0.015**
Central DBP (mmHg)	77.6 ± 8.8	67.8 ± 7.3	**0.009**
Central MAP (mmHg)	87.7 ± 9.3	77.5 ± 6.9	**0.007**
Resting HR (beats min^−1^)	72.0 ± 9.6	52.5 ± 9.9	**< 0.001**
$\dot{V}$O_2peak_ (ml kg^−1^ min^−1^)	34.7 ± 4.4	54.6 ± 13.1	**< 0.001**

Data are expressed as mean ± standard deviation from two-tailed independent samples *t*-tests or Mann–Whitney U-tests, with *P*-values below 0.05 bolded. Variances were equal between groups for all variables except for age.

BMI, body mass index; SBP, systolic blood pressure; DBP, diastolic blood pressure; HR, resting heart rate; $\dot{V}$O_2peak_, maximal aerobic (cardiorespiratory) fitness.

**Table 5:** Results of sensitivity analysis performed on male athlete status of an individual testing cohort demographic and cardiovascular health characteristics as well as LCN2 concentration.

Dependent variable	Cox & snell R^2^	Variables	*β*	*β* Std. Error	*P* value	Overall model
Athlete status	0.676	BMI	N/A	N/A	0.089	log(p/1 − p) = 0.401(Age) + 0.743(VO_2peak_)
		**Age**	**0.401**	**0.148**	**0.007**	
		**VO**_**2peak**_	**0.743**	**0.254**	**0.003**	
		LCN2 Concentration	N/A	N/A	0.956	

Athletes were assigned as 1 in the dataset, while controls were assigned as 0. The overall model describes the relationship between significant predictors of athlete status and the predicted log odds of the individual tested being an athlete. The model correctly predicted athlete status in 90.5% of individuals in our dataset.

BMI, body mass index; SBP, systolic blood pressure; DBP, diastolic blood pressure; LCN2, lipocalin-2; $\dot{V}$O_2peak_ maximal aerobic (cardiorespiratory) fitness.

We also performed sensitivity analyses to determine the influence of LCN2 on SBP, DBP, central SBP and central DBP (Tables 6, 7). While a generalised model predicting SBP did not show LCN2 concentration as a significant factor, models predicting DBP, central SBP, and central DBP found that LCN2 concentration was a significant predictor (*P* < 0.05, Tables 6, 7). All models explained 21.1% to 29.2% of the variability of the predicted variable around its mean as indicated by their r^2^ values. The *β* values associated with LCN2 concentration were small and ranged between 0.105 and 0.128, suggesting that the effect of LCN2 on these factors was limited in scale.

**Table 6:** Sensitivity analysis performed on blood pressure testing cohort demographic characteristics and LCN2 concentration.

Dependent variable	Adjusted R^2^	Variables	*β*	*β* Std. Error	*P* value	Overall model
SBP	0.292	**Sex**	**9.938**	**1.951**	**< 0.0001**	89.605 + 9.938 (Male) + 1.125*BMI
		**BMI**	**1.125**	**0.285**	**< 0.0001**	
		Age	N/A	N/A	0.1831	
		Athlete Status	N/A	N/A	0.7227	
		LCN2 Concentration	N/A	N/A	0.1548	
DBP	0.257	Sex	N/A	N/A	0.322	33.671 + 1.291*BMI + 0.128*LCN
		**BMI**	**1.291**	**0.243**	**< 0.0001**	
		Age	N/A	N/A	0.6438	
		Athlete Status	N/A	N/A	0.3916	
		**LCN2 Concentration**	**0.128**	**0.04**	**0.0018**	
Central SBP	0.211	**Sex**	**5.907**	**2.061**	**0.0052**	74.087 + 0.948*BMI + 5.907 (Male) + 0.105*LCN—3.382 (Athlete)
		**BMI**	**0.948**	**0.362**	**0.0105**	
		Age	N/A	N/A	0.1996	
		**Athlete Status**	− **3.382**	**2.163**	**0.1217**	
		**LCN2 Concentration**	**0.105**	**0.05**	**0.0385**	
Central DBP	0.229	Sex	N/A	N/A	0.309	35.927 + 1.27*BMI + 0.114*LCN
		**BMI**	**1.27**	**0.262**	** < 0.0001**	
		Age	N/A	N/A	0.91	
		Athlete Status	N/A	N/A	0.155	
		**LCN2 Concentration**	**0.114**	**0.041**	**0.007**	

Significant contributors to the model are bolded, with their specific contributions shown in the overall model column.

BMI, body mass index; SBP, systolic blood pressure; DBP, diastolic blood pressure; LCN2, lipocalin-2.

**Table 7:** Sensitivity analysis performed on $V$O_2peak_ testing cohort demographic characteristics and LCN2 concentration. Significant contributors to the model are bolded, with their specific contributions shown in the overall model column.

Dependent variable	Adjusted R^2^	Variables	*β*	*β* Std. Error	*P* value	Overall model
$V$O_2peak_	0.687	**Sex**	**7.817**	**1.471**	** < 0.0001**	59.174 + 7.817 (Male) + 13.907 (Athlete) – 0.501*BMI – 0.3*Age
		**BMI**	− **0.501**	**0.271**	**0.068**	
		**Age**	− **0.3**	**0.061**	** < 0.0001**	
		**Athlete Status**	**13.907**	**1.61**	** < 0.0001**	
		LCN2 Concentration	N/A	N/A	0.346	

$\dot{V}$O_2peak_ maximal aerobic (cardiorespiratory) fitness.

**Figure 3:**
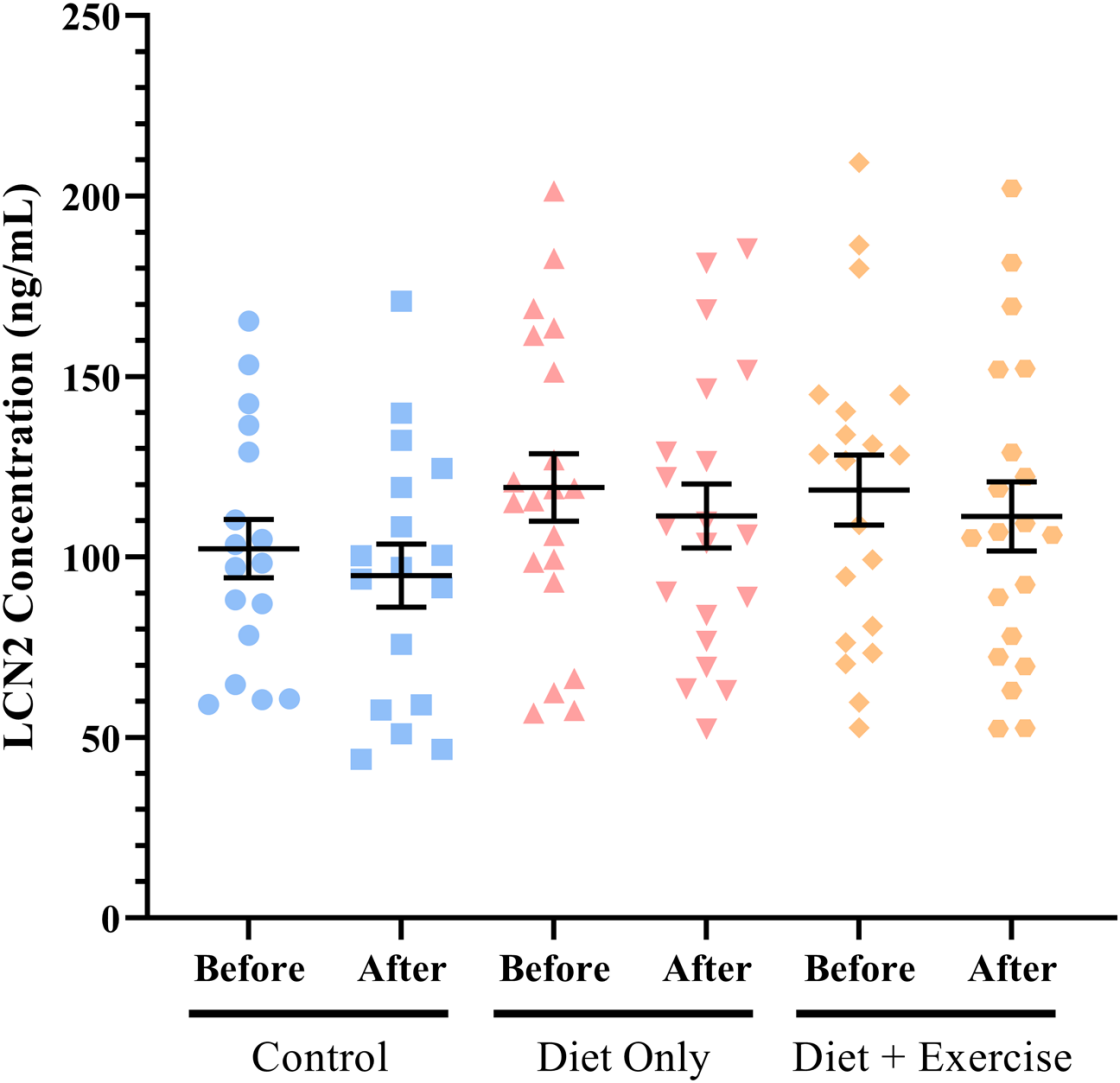
Plasma lipocalin-2 (LCN2) protein levels before and after a randomised control trial. There was no difference in LCN2 levels between control (n = 17), dietary intervention (n = 20), or dietary intervention and exercise arms (n = 20) (*P* = 0.37). Comparisons of initial and post-intervention LCN2 concentrations also showed no significant changes (*P* = 0.47, *P* = 0.60, *P* = 0.51 for comparisons in the control, diet intervention, and dual intervention group respectively). Data are expressed as mean ± S.E.M.

In the randomised control trial, we compared the difference between time points in all groups in the intervention study. We had previously reported that the diet + exercise group was the only group to have a significant increase in $\dot{V}$O_2peak_, which validates the exercise intensity methodology^30^. No groups differed significantly from one another in the context of age, sex, plasma LCN2 content, or cardiovascular factors, although the BMI between groups differed significantly post-intervention (*P* = 0.0008, Table 8). While LCN2 protein concentration did not differ substantially between any of the intervention groups (all *P* > 0.05, Fig. 3) or within each group when comparing initial and post-intervention expression (all *P* > 0.05), the diet and exercise intervention successfully reduced BMI (*P* = 0.04) compared to the control group (Table 8). This change was not reflected in the cardiovascular health indicators of the intervention groups, as both SBP and DBP remained similar between the control and integrated diet-exercise groups. We found that LCN2 levels remained stable over the 12-week interventions in all groups (Table 8). Furthermore, initial and post-intervention LCN2 levels were significantly correlated with each other between all groups (Table 9). Plasma CRP levels pre-intervention and post-intervention were also significantly correlated with each other, but did not correlate with plasma LCN2 levels (Table 10).

**Table 8:** Demographic and cardiovascular characteristics of intervention groups.

	Control (n = 17)		Diet intervention (n = 20)		Diet + exercise interventions (n = 20)		*P *values	
	Pre-intervention	Post-intervention	Pre-intervention	Post-intervention	Pre-intervention	Post-intervention	Pre-intervention	Post-intervention
Age (years)	53.90 ± 5.65		55.46 ± 6.25		54.28 ± 4.43		0.6629	
Men/Women (n)	11/6		12/8		12/8		0.9487	
BMI (kg/m^2^)	33.10 ± 3.69	33.38 ± 3.83	32.27 ± 3.81	29.77 ± 3.59	31.82 ± 3.73	28.65 ± 3.52*	0.5862	**0.0008**
SBP (mmHg)	154.52 ± 25.73	153.38 ± 22.40	157.75 ± 25.64	153.01 ± 26.11	155.07 ± 24.17	144.98 ± 19.97	0.9157	0.4585
DBP (mmHg)	82.54 ± 10.44	78.68 ± 10.57	86.96 ± 11.57	81.69 ± 13.83	87.06 ± 10.06	77.91 ± 10.59	0.3812	0.5944
HR (beats min^−1^)	61.98 ± 9.34	61.26 ± 6.98	64.05 ± 8.07	59.11 ± 6.81	58.97 ± 6.96	57.21 ± 8.45	0.1486	0.2839

Data are expressed as mean ± standard deviation from one-way ANOVAs comparing values between the three groups, with *P* values below 0.05 bolded.

BMI, body mass index; SBP, systolic blood pressure; DBP, diastolic blood pressure; HR, resting heart rate.

*Tukey’s honest significance test comparing BMIs from the control group to the diet and exercise intervention, resulting from a mixed ANOVA, with *P* = 0.04.

**Table 9:** Pearson’s correlations between measured plasma LCN2 concentrations pre- and post-interventions per group.

Intervention group	r	FDR-adjusted *q*-value
Control	0.708	**0.001**
Diet intervention	0.703	**0.001**
Diet and exercise intervention	0.777	** < 0.001**
All groups combined	0.743	** < 0.001**

Data are from two-tailed Pearson’s correlations, with *q*-values under 0.05 bolded.

FDR, False Discovery Rate.

**Table 10:** Pearson’s correlations between measured plasma CRP (mg/l) concentrations pre- and post-interventions per group, as well as correlations with LCN2.

Correlations	Intervention Group	r	FDR-adjusted *q*-value
Pre- and post-intervention plasma CRP correlations	Control	0.557	**0.032**
	Diet intervention	0.629	**0.008**
	Diet and exercise intervention	0.786	**0.0003**
	All groups combined	0.675	**< 0.0001**
Post-intervention CRP and LCN2 correlations	Control	0.411	0.1616
	Diet intervention	− 0.220	0.468
	Diet and exercise intervention	0.111	0.7429
	All groups combined	0.008	0.952

Data are from two-tailed Pearson’s correlations, with *q*-values under 0.05 bolded.

FDR, False Discovery Rate; LCN2, Lipocalin-2; CRP, C-reactive protein.

## Discussion

Emerging evidence proposes that LCN2 is not merely a biomarker of cardiac and renal diseases, but has a role in modulating chronic inflammation and other processes that can lead to these diseases^19,31,32^. Chronic inflammation is usually associated with poor diet, low physical activity and higher BMI, which are risk factors for all CVDs. Here we showed that LCN2 is not modulated by exercise in two independent cohorts. Moreover, a three-month intervention with the DASH diet, which is associated with lower inflammation, was not able to lower levels of LCN2. Finally, LCN2 levels were highly correlated three months apart, independently of the type of intervention and BMI changes. Together, our findings suggest that LCN2 is stable over time and is not modulated by exercise nor diet in healthy or overweight/obese people.

When comparisons were drawn between the control and athlete groups, we found no significant difference in LCN2 levels, even though conventional cardiovascular health markers were markedly different between the groups. Athletes had a significantly healthier cardiovascular profile, consistent with previous studies which have found athletes to have longer telomeres and lower general and CVD mortality^33–38^. Interestingly, while a recent study suggested that LCN2 expression is sex-specific in mice, we could not pinpoint any significant differences of plasma LCN2 expression in humans between athletes and controls in male or female groups^39^. The ability of repeated exercise to rapidly attenuate the initially-seen physiological stress response has been well reported, even when the exercise is scaled with the participant’s increasing fitness^40,41^. A strong acute phase response may have initially occurred in newer athletes which quickly diminished over their time spent training, causing plasma LCN2 expression to stabilise. In addition, not all components of the acute phase response may be activated by exercise, instead creating a diminished acute phase response. The literature surrounding this topic suggests that partial activation of the acute phase response after extreme endurance exercise is common^42^. It is possible that circulating LCN2 is unaffected by physiological stress associated with long-term, repeated endurance training, and, therefore, would be unchanged in athletes when compared to apparently healthy controls. However, multiple studies have found significant changes in post-aerobic exercise serum and plasma LCN2 concentrations, which suggests that the acute phase response exhibited in response to moderate to intense aerobic exercise is sufficient to cause expression of LCN2^43–45^. Another possibility is that LCN2 is genetically determined, and is minimally influenced by lifestyle factors such as exercise. Indeed, in a previous study we found that the single nucleotide polymorphism rs13297295 located near the gene coding *LCN2* was a cis-expression quantitative gene locus for LCN2 levels in a large cohort, suggesting that plasma LCN2 levels are primarily under genetic control^19^.

LCN2 has been proposed as an inflammatory marker for obesity, hyperglycaemia and insulin resistance, and has also been found to drive left ventricular pathological cardiac hypertrophy^19,46^. Due to LCN2 being under genetic control of the NF-kβ and MAPK pathway, both involved in chronic low-grade inflammation found in obese and hypertensive individuals, circulating LCN2 expression is possibly stable for as long as inflammation status remains relatively unchanged. Our data, however, suggested that plasma LCN2 levels are resistant to modification by short-term interventions known to decrease inflammation, such as diet or exercise, and levels were stable over a period of three months. Indeed, there was no change in LCN2 with a decrease in BMI. Whether lean tissue mass is associated with LCN2 remains to be determined. Although our sensitivity analyses also showed that LCN2 was associated with SBP and DBP, the ability of LCN2 to predict blood pressure values was low and other traditional risk factors, such as sex and BMI, were more important. Furthermore, the immutable nature of plasma LCN2 in response to the exercise or diet regimens and its lack of correlation to various cardiovascular health markers lends credence to the applicability of LCN2 as a potential biomarker for CVD, as it may be directly influenced by the development of CVD as opposed to being influenced by non-disease related cardiovascular health markers (e.g. diet and $\dot{V}$O_2peak_). This is further supported by previous research, where LCN2 has been found to be a predictor of cardiovascular mortality over an 11 year longitudinal study^47^.

Interestingly, we found that decreases in BMI nor changes in plasma CRP over the three month period were not associated with changes in LCN2 plasma concentration. The association of circulating LCN2 with BMI and other cardiovascular health markers has not been elucidated clearly in the literature, with multiple studies conflicting on whether LCN2 and BMI change in tandem^48–51^. LCN2 levels, however, are correlated with obesity and hyperglycaemia, both of which have been shown to induce and promote a chronic low-grade inflammatory state^52–54^. Our results support the hypothesis that BMI and LCN2 are not intrinsically linked together, and that independent modulation of either variable is possible. Plasma LCN2 levels, however, may be attenuated over longer time periods as participants recover from obesity and concomitant chronic low-grade inflammation.

One limitation of this study is the singular type of exercise chosen as the intervention. Many aspects of exercise were controlled in our study, such as duration, frequency, intensity, and mode. However, other regimens such as high intensity interval training (HIIT) or resistance training have also shown to be effective exercises for cardiovascular health, with HIIT shown to improve both blood pressure and cardiovascular fitness^55^. Moreover, overweight/obese subjects may display chronotropic incompetence and as such exercise intensity based on percentage of maximal heart rate is not always ideal. However, we still observed a significant increase in $\dot{V}$O_2peak_ in the diet + exercise group only^30^. Furthermore, as LCN2 levels are positively correlated with left ventricular hypertrophy, longitudinal measurements of LCN2 and cardiac size in long-term athletes may yield interesting results as exercise induces physiological hypertrophy of the heart^19,56^. Additionally, while our study measured plasma LCN2, LCN2 content in skeletal muscle or cardiac tissue was not measured. Due to LCN2 being linked to the MAPK pathway and because the pathway is heavily involved in muscle regeneration, it is possible that skeletal muscle LCN2 content is influenced by acute and long-term exercise^57–59^. Our study had several strengths including the use of two independent cohorts, one which was a cross-sectional cohort and a randomised control trial. Furthermore, it is possible that our intervention timeframe of 12 weeks was insufficient to significantly change the inflammatory phenotype that had been built over a long period in overweight or obese individuals. While we would expect to still see a change in plasma LCN2 expression within the intervention groups due to the positive correlations between BMI and LCN2 expression, along with correlations between BMI and inflammatory markers described in the current literature, the change may not have been large enough to elucidate significance^46,60–62^.

In conclusion, LCN2 levels were not different between athletes and controls, nor were they modified after three months of interventions with diet and exercise. While LCN2 was a significant variable included in models predicting blood pressure in athletes or controls, the low *β* values for each suggest that LCN2 has minimal effects. Importantly, our data shows that circulating LCN2 plasma concentration is not dependent on BMI, and is stable over a period of three months. Pre-intervention LCN2 levels were found to be strongly correlated with post-intervention expression. Further research may elucidate a molecular pathway between LCN2 expression and multiple renal and CVDs.

## Methods

All participants gave written informed consent. The studies were approved by the Alfred Hospital and the Federation University Australia Human Research Ethics Committees, and followed the Declaration of Helsinki.

### Athlete and control participants

Endurance athletes and control subjects involved in a cross-sectional analyses were described elsewhere^63,64^. Briefly, 52 endurance athletes (cyclists, runners and triathletes) and 48 controls (aged 18–55) were recruited at Federation University Australia from local advertisements, social media and word of mouth (Table 1). Athletes engaged in vigorous endurance exercise at least three times per week, for a minimum of one year. Endurance athletes were cyclists, triathletes, middle- or long-distance runners, and ultra-marathon runners at state through to international level. The apparently healthy controls were recreationally active but were not engaged in any structured aerobic or resistance exercise training. All subjects were non-smoking, free from any age-related chronic diseases and not taking any medications. The controls were recreationally active but were not engaged in any structured aerobic or resistance exercise training. All BP assessments were conducted in the AM following an overnight fast. Brachial and central BP was measured with participants in a seated position after a 10-min rest period using the SphygmoCor Device (AtCor Medical, Australia). Blood pressures were calculated from the average of three measurements, recorded one minute apart^63^.

### Diet and exercise intervention participants

Overweight and obese middle-aged (45–65 year old) men and post-menopausal women who fulfilled Adult Treatment Panel III criteria for metabolic syndrome (MetS) were recruited at the Alfred Hospital through newspaper advertisements (clinicaltrials.gov registration number NCT00163943, first registration 14/09/2005), as previously reported^30^. All subjects were sedentary and not taking any medication while being free from non-communicable diseases, but were overweight or obese (waist circumference: ≥ 102 cm for men and ≥ 88 cm for women). None of the participants had type 2 diabetes or a history of secondary hypertension, cardiovascular, cerebrovascular, renal, liver, thyroid disease, or drug use known to affect these parameters. Supine blood pressure was measured in the clinic as the average of five readings after a 5-min rest (Dinamap, Model 1846SX; Critikon, Tampa, FL). Body weight was measured in light indoor clothes without shoes, using a digital scale. Waist circumference was measured at the midpoint between the lowest rib and iliac crest and hip circumference at the level of the greater trochanters. Stratified randomization by sex and hypertension status in blocks of six was used to allocate subjects to one of three groups. Participants were randomly allocated into three arms of a 12-week intervention: control arm (no intervention), diet arm, or exercise and diet arm. The diet and exercise intervention protocol has also been reported, which consisted of 40 min of bicycle riding on alternating days over 12 weeks^30^. Exercise was moderate, with participants reaching 65% of a predetermined maximum heart rate throughout. Maximum work capacity and maximum oxygen consumption were measured during a "sprint" exercise test. The exercise test was supervised by a cardiologist and was performed on a bicycle ergometer, commencing at zero work rate. Each minute the work rate was increased 20 W until any further increase was prevented by fatigue. The 14-day menu plans, recipes, and prepared meals followed a modified Dietary Approaches to Stop Hypertension (DASH) diet, and was composed of 30% fat (6% polyunsaturated, 15% monounsaturated, and 9% saturated), 22% protein, and 48% carbohydrate as previously described^65,66^. They were provided with a 14-day menu plan and recipes, and prepared meals in their homes. High sensitivity CRP was measured in fasting samples by immunoturbidimetric assay.

Basal energy requirements were calculated for each individual by indirect calorimetry and energy intake was reduced by 600 cal per day as previously described. Subjects were provided with 14-day menu plans and recipes and prepared meals in their homes. Participants attended at two weekly intervals for dietary counselling. Compliance was assessed by prospective 4-day diet records, which were analysed using Australian Food Composition Tables (FoodWorks Professional Version 3.02; Xyris Software, Highgate Hill, Australia https://xyris.com.au/products/foodworks-10-professional/). Sodium, potassium, and protein intake were quantified by 24-h urine collections.

Exercise training comprised 40 min bicycle riding on alternate days at a moderate intensity of 65% of predetermined maximum heart rate^67^. This corresponded to target heart rates within the range 120 –145 bpm during exercise. Workload was increased as necessary to maintain target heart rate. Once a week, exercise was performed under supervision in our clinical laboratory at the Alfred Hospital Heart Centre. Other sessions were performed at the subjects’ homes using provided exercise bicycles and heart rate monitors. Subjects kept records of average heart rate during each exercise session. Compliance was assessed by review of heart rate records and at the end of the intervention from measures of maximal oxygen consumption during a continuous incremental cycle ergometry protocol, during which workload was increased by 20 W/min.

Participants in the control group were instructed to maintain their usual dietary and exercise habits. They attended our laboratory at the Heart Centre every 3 weeks for body weight and blood pressure assessment.

### Plasma measurement of LCN2

All participants gave a fasted (12 h) blood sample collected from the antecubital vein using standard phlebotomy procedures. A resting blood sample was collected with participants seated. Subjects were asked to refrain from alcohol, caffeine and exercise training at least 24 h before blood donation. Plasma was isolated from peripheral blood that was collected into EDTA tubes and stored at − 20 °C. Plasma LCN2 abundance was measured in duplicates by enzyme-linked immunosorbent assay (ELISA) run in duplicate using the Human NGAL ELISA Kit (ElabScience, E-EL-H0096) according to the supplier’s guidelines. Intra-assay coefficients of variability were calculated and only samples with less than 15% variability were accepted. Overall inter-assay coefficient of variability was 5.4–9.7%.

### Statistical analyses

Outliers were removed using the robust regression and outlier removal algorithm in GraphPad Prism (version 8.1.2, GraphPad Software, CA, USA https://www.graphpad.com/scientific-software/prism/), using a Q value of 2%. The ROUT algorithm is based on a nonlinear regression to fit an outlier-independent curve, which is then used to identify and remove outliers. Two controls and one athlete were excluded from the athlete and control cohorts based on the ROUT algorithm, with LCN2 concentrations of 1.638 ng/mL, 147.714 ng/mL, and 1.514 ng/mL. No participants were found to be outliers for the intervention cohorts. Data was first tested for normality using the D’Agostino-Pearson test for normality, and comparisons between controls and athletes were performed using two-tailed independent samples t-tests or Mann–Whitney U-tests for continuous variables, and chi-squared tests for categorical variables (i.e. biological sex) in SPSS (version 24, IBM, NY, USA https://www.ibm.com/au-en/analytics/spss-statistics-software), and the equalities of variances of variables between groups was computed using Levene’s test. Correlation between LCN2 levels and cardiovascular heath indicators (including systolic and diastolic blood pressure, mean arterial pressure, and central blood pressures) were calculated using Pearson’s correlation, with *P*-values further corrected for multiple comparisons using the ‘p.adjust’ function with the false discovery rate (FDR) correction argument in R (version 3.6.1, R Foundation, Vienna, Austria https://www.r-project.org/). Sensitivity analysis of LCN2 levels and other significant cardiovascular factors was performed through usage of a forward stepwise linear regression analysis in SPSS, using the probability of *F* as an entry and exit factor at 0.15 and 0.20 respectively. An additional sensitivity analysis in the form of a forward stepwise binary logistic regression model was performed for the entire cohort as well as sex-specific subgroups, testing the predictability of athlete status from LCN2 concentration, cohort characteristics, and cardiovascular health indicators. The R^2^ (adjusted R^2^ values for linear regression models, Cox-Snell R^2^ values for binary logistic regression models), *β*, and *P*-values were reported alongside the general model equations.

Comparisons between controls, diet, or diet and exercise arms were performed using a mixed analysis of variance (MANOVA), with the comparison between the baseline and post-intervention observations as the within-group effect and the intervention type as the between-group effects. Multiple comparisons were subsequently performed using Tukey’s honest significance test. Correlations between initial and post-intervention plasma LCN2 concentration were performed using a Pearson’s correlation, with r and FDR-adjusted *q*-values reported. Data are reported as means, with error bars representing standard error of the mean (S.E.M.) or standard deviation (SD). The mean and standard deviation (S.D.) are reported for cohort characteristics. *P* or FDR *q*-values below 0.05 were considered significant.
